# Perception of sleep-related disturbs by parents/guardians of patients with respiratory disease treated at the children's asthma prevention program (PIPA) - Uruguaiana, RS, Brazil

**DOI:** 10.1186/1939-4551-8-S1-A10

**Published:** 2015-04-08

**Authors:** Marilyn Urrutia Pereira, Dirceu Sole, Jennifer Avila

**Affiliations:** 1 Programa Infantil De Prevencao Asma, Brazil; 2 Brazilian Society, Brazil

## Background

Inadequate sleep in children has been blamed for compromising cognitive function, school performance, among others. These changes also compromise the quality of life of children as well as parents. Sleep impairment has been described in 10% to 75 % of children with persistent and severe diseases. The CSHQ is one of the instruments used to assess sleep quality in children. The objective of this study was to assess sleep behavior of children and adolescents followed at PIPA according to disease presented: asthma isolated or associated with other allergic diseases.

## Methods

The validated version of CSHQ in Brazilian Portuguese was answered by parents of children and adolescents (n=296, 6 mo to 17 yrs) when admitted in PIPA. The scores of each of the 33 questions were entered into an Excel spreadsheet to enable the achievement of the eight subclasses scores and the total score. Patients were divided into two groups: asthma alone (AA), and asthma associated with allergic rhinitis and atopic dermatitis (A+AR+AD). Total CSHQ score was correlated with body mass index (BMI). Statistical analysis was performed by Student t test and Spearman correlation with significance level of p less than 5%.

## Results

There were no differences regarding mean age (months) of patients in AA and A+AR+AD groups (84.0 vs. 80.9, respectively). Total CSHQ score of group A (84.6±7.0) was significantly higher than A+AR+AD (82.7±7.2). However, there were no significant differences concerning the subscales that compose the CSHQ. Although the mean score of patients with AA was higher than those with A+AR+AD, the difference was significant only when considering the total score. The correlation between BMI with total CSHQ score was not significant.

## Conclusions

Although there were no significant differences between the studied groups, the CSHQ scores observed were higher than those obtained during the validation of the questionnaire. Monitoring of sleep in patients with respiratory allergy is an important tool in the evaluation of their treatment.

